# Fingolimod (FTY720) therapy in Japanese patients with relapsing multiple sclerosis over 12 months: results of a phase 2 observational extension

**DOI:** 10.1186/1471-2377-14-21

**Published:** 2014-01-29

**Authors:** Jun-ichi Kira, Yasuto Itoyama, Seiji Kikuchi, Qi Hao, Takayoshi Kurosawa, Kazuo Nagato, Isao Tsumiyama, Philipp von Rosenstiel, Lixin Zhang-Auberson, Takahiko Saida

**Affiliations:** 1 Department of Neurology, Neurological Institute, Graduate School of Medical Sciences, Kyushu University, Fukuoka, Japan; 2 National Center Hospital, National Center of Neurology and Psychiatry, Tokyo, Japan; 3 Hokkaido Medical Center, National Hospital Organization, Sapporo, Japan; 4 Institute of Neurotherapeutics, Kyoto, Japan; 5 Novartis Pharma KK, Tokyo, Japan; 6 Mitsubishi Tanabe Pharma Corporation, Tokyo, Japan; 7 Novartis Pharma AG, Basel, Switzerland; 8 Department of Neurology, Kyoto Min-Iren-Central Hospital, Kyoto, Japan; 9 Kyoto University Hospital, Kyoto, Japan

**Keywords:** Aquaporin 4, Fingolimod, Longitudinally extensive spinal cord lesions, Multiple sclerosis, Phase 2 study

## Abstract

**Background:**

A 6-month phase 2 study of fingolimod demonstrated efficacy and safety in Japanese patients with relapsing-remitting multiple sclerosis (MS). Here we report a 6-month observational extension that evaluated efficacy and safety in patients who received fingolimod continuously for 12 months or who switched from placebo to fingolimod.

**Methods:**

Of 147 patients who completed the 6-month core study, 143 entered the extension. Those originally randomized to placebo were re-randomized to fingolimod 1.25 mg or 0.5 mg. During the extension, all patients were switched to open-label fingolimod 0.5 mg.

**Results:**

Magnetic resonance imaging (MRI) and relapse outcomes were maintained or improved in patients treated with fingolimod for 12 months versus those treated for 6 months. No new safety events were reported over 12 months of treatment. Infections occurred in similar proportions of continuously treated and switched patients, while cardiac and liver adverse events occurred in fewer continuously treated than switched patients. Four patients were aquaporin-4 (AQP4) antibody-positive, three of whom showed rapid disease exacerbations within 10 days of fingolimod initiation.

**Conclusion:**

Continuous fingolimod treatment for up to 12 months was associated with maintained or improved efficacy and a manageable safety profile, consistent with that previously seen. Results in a small number of patients suggest lack of benefit in AQP4 antibody-positive patients. Meaningful statistical interpretation was limited by the small sample size in each treatment group, owing to the number of patients who completed the core study.

**Trial registration:**

ClinicalTrials.gov NCT00670449

## Background

Fingolimod (FTY720) 0.5 mg once daily is the first oral therapy approved for relapsing multiple sclerosis (MS) in many countries, including the USA [1], for relapsing-remitting MS (RRMS) with high disease activity in the European Union [2], and for the treatment of MS in Japan [3]. Fingolimod binds to sphingosine 1-phosphate receptors (S1PRs) on lymphocytes leading to retention of circulating lymphocytes in the lymph nodes. This reversible reduction in the number of peripheral blood lymphocytes is postulated to be mechanistically important in MS, decreasing the recirculation of autoreactive lymphocytes and preventing their infiltration into the central nervous system [4,5].

Global phase 2 and 3 clinical studies have established a favorable efficacy and safety profile for fingolimod in predominantly Caucasian populations with MS [6-9]. A 6-month, phase 2, registration study (ClinicalTrials.gov Identifier NCT00537082) in Japanese individuals demonstrated that fingolimod was associated with reduced inflammatory brain lesion activity and relapse rate compared with placebo [10].

This phase 2 extension study aims to assess the long-term efficacy, safety and tolerability of fingolimod in Japanese patients with relapsing MS. Here we report 12-month efficacy and safety data in individuals continuously receiving fingolimod and in those whose treatment was switched to fingolimod during months 7–12 from placebo during months 0–6 (termed the placebo-fingolimod group). The effects of delaying the initiation of fingolimod therapy can be assessed by comparing patients continuously treated with fingolimod from month 0 and patients switched to fingolimod 6 months later.

## Methods

### Patients

Patients with relapsing MS who had completed 6 months of treatment in the core study were eligible for the extension study. Eligibility criteria for the core study have been described in detail elsewhere [10]. In brief, patients had to be aged 18–60 years and have a diagnosis of MS according to the revised McDonald criteria [11] with recent relapses or at least one baseline gadolinium (Gd)-enhancing T1 lesion on magnetic resonance imaging (MRI). Patients with longitudinally extensive spinal cord lesions (LESCLs) of at least three segments (a marker of neuromyelitis optica [NMO]) at screening were excluded. Those who completed the core study were invited to give informed consent to enter the extension phase unless a study investigator assessed them to be medically ineligible.

### Study oversight and design

Information about study oversight has been published previously [10]. The study (ClinicalTrials.gov Identifier, NCT00670449) was conducted in accordance with the International Conference on Harmonisation Guidelines for Good Clinical Practice [12] and the Declaration of Helsinki [13] and was overseen by an independent Data Safety Monitoring Board. Prior to conducting the study, the Institutional Review Board at each participating medical centre provided ethical approval of the study protocol as well as the case report forms, patient information and informed consent forms. In the core study, patients were randomly assigned in a 1:1:1 ratio to receive once-daily fingolimod capsules, 0.5 mg or 1.25 mg, or matching placebo for 6 months according to the procedures previously published [10]. After entering the extension study, patients either continued to receive their initial dose of fingolimod (0.5 mg or 1.25 mg) or were re-randomized 1:1 from placebo to fingolimod 0.5 mg or 1.25 mg under double-blind conditions. Late in the study, emerging efficacy and safety data from the phase 3 clinical trial program [6,8] indicated a more favorable benefit-risk profile for fingolimod 0.5 mg than for the higher fingolimod dose. Based on these results, all patients who had yet to enter the extension study and who had received fingolimod 1.25 mg or placebo in the core study, and patients who were continuing to receive fingolimod 1.25 mg in the extension study, had their treatment switched to fingolimod 0.5 mg. The mean time to switch from administration of the first dose in the core study was 399 days for the fingolimod 1.25 mg group and 344 days for the placebo-fingolimod 1.25 mg group. The majority of patients switched by 12 months from core study start (fingolimod 1.25 mg, 25/46 [54.3%] patients; placebo-fingolimod 1.25 mg, 12/23 [52.2%] patients).

### Study endpoints and procedures

Key efficacy variables were MRI inflammatory activity (proportions of patients free of Gd-enhancing T1-weighted or new/newly enlarged T2-weighted MRI lesions and numbers of Gd-enhancing T1-weighted or new/newly enlarged T2-weighted MRI lesions) and relapse activity (proportions of patients free of relapses over 12 months, time to the first confirmed relapse up to 12 months and annualized relapse rate [ARR]).

Standardized MRI images were obtained as described previously [7] at screening and months 3, 6, 9 and 12. Expanded Disability Status Scale (EDSS) scores were determined at months 6, 9 and 12. Clinical and safety assessments were conducted on day 1 of entry into the extension study and at months 6.5, 7, 8, 9, 10, 11 and 12. Vital signs were monitored for at least the first 6 hours after first-dose administration of fingolimod. Anti-aquaporin-4 (AQP4) antibody test results were collected retrospectively from medical records of patients who consented to provide the data during the study.

### Statistical methods

The analysis population for MRI endpoints included all patients who received at least one dose of study drug during the extension study and had at least one valid MRI scan during the extension study at month 9 or later (MRI analysis population). The full analysis set (all patients who received at least one dose of study drug during the extension phase) was used for relapse and EDSS endpoints (clinical analysis population) and safety endpoints (extension safety population). Efficacy and safety endpoint data were summarized according to the four groups (fingolimod 1.25 mg, fingolimod 0.5 mg, placebo-fingolimod 1.25 mg and placebo-fingolimod 0.5 mg). The change in dose to fingolimod 0.5 mg during the study for patients initially randomized to fingolimod 1.25 mg in either the core study or extension study precluded the possibility of meaningful formal statistical comparisons. Therefore, analyses were summarized descriptively.

## Results

### Study population

**Figure 1:**
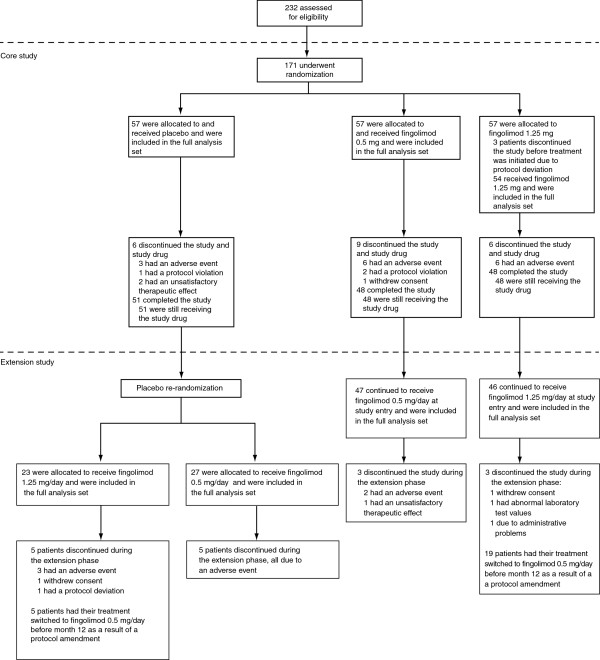
Enrollment, randomization and follow-up of study patients.

A total of 171 patients were randomized in the core study across 43 centers in Japan [10]; 147 completed the trial [10] and 143 agreed to enter the extension phase (Figure 1). The extension began in April 2008 and all of the patients entered into the study had completed the month 12 visit by August 2010. The 6-month extension phase was completed by 127 patients. Adverse events (AEs) accounted for most discontinuations (Figure 1). The baseline demographics of the extension randomized population were similar across treatment groups (Table 1). Baseline MS disease characteristics of the extension randomized population appeared to be generally similar across treatment groups (Table 1) with the exception of mean number of relapses in the previous 1 or 2 years and the proportion of patients free of Gd-enhancing lesions.

**Table 1:** Baseline demographics and clinical characteristics of patients at entry to core study (extension randomized population)

**Characteristic**	**Fingolimod 0.5 mg (n = 47)**	**Fingolimod 1.25 mg (n = 46)**	**Placebo-fingolimod 0.5 mg (n = 27)**	**Placebo-fingolimod 1.25 mg (n = 23)**
**Patient demographic**				
Age, years				
Mean (SD)	34.9 (9.0)	35.7 (8.8)	34.2 (9.1)	35.5 (8.4)
Median (range)	34.0 (19–52)	36.0 (18–55)	34.0 (18–52)	34.0 (21–51)
Female, n (%)	33 (70.2)	31 (67.4)	19 (70.4)	14 (60.9)
BMI, kg/m^2^				
Mean (SD)	21.8 (3.2)	22.0 (3.9)	21.0 (2.6)	20.7 (3.1)
Median (range)	21.5 (15.1–32.6)	21.1 (18.1–36.2)	20.8 (15.0–26.2)	20.2 (13.8–28.8)
**Disease characteristic**				
Clinical pattern of MS, n (%)				
Relapsing-remitting	44 (93.6)	45 (97.8)	27 (100.0)	23 (100.0)
Secondary progressive	3 (6.4)	1 (2.2)	0 (0.0)	0 (0.0)
Duration of MS since first symptom, years				
Mean (SD)	8.2 (6.6)	7.6 (5.5)	8.4 (8.1)	8.4 (7.2)
Median (range)	6.4 (1–26)	6.2 (0–21)	5.4 (1–27)	5.9 (1–24)
Number of relapses within previous year				
Mean (SD)	1.4 (0.9)	1.5 (1.0)	2.1 (2.1)	1.4 (0.7)
Median (range)	1.0 (0–3)	1.0 (0–4)	2.0 (1–12)	1.0 (0–3)
Number of relapses within previous 2 years				
Mean (SD)	2.1 (1.3)	2.2 (1.6)	3.2 (4.0)	2.5 (1.4)
Median (range)	2.0 (0–7)	2.0 (0–6)	2.0 (1–22)	2.0 (0–5)
EDSS score				
Mean (SD)	2.4 (1.9)	1.9 (1.7)	1.9 (1.6)	2.4 (1.6)
Median (range)	2.0 (0.0–6.0)	2.0 (0.0–6.0)	1.5 (0.0–5.0)	2.0 (0.0–5.5)
**MRI characteristics**				
Patients free of Gd-enhancing lesions				
n (%)	28 (59.6)	22 (47.8)	13 (48.1)	17 (73.9)
Number of Gd-enhancing lesions				
Mean (SD)	1.0 (1.59)	1.7 (2.42)	1.7 (2.45)	0.7 (1.49)
Median (range)	0.0 (0–5)	1.0 (0–9)	1.0 (0–9)	0.0 (0–5)
Number of T2 lesions				
Mean	30.3 (22.83)	34.6 (24.15)	28.9 (23.22)	33.3 (23.11)
Median (range)	24.0 (4–100)	29.5 (5–119)	23.0 (3–98)	35.0 (1–91)
**MS medication history**				
Treatment-naïve patients, n (%)	16 (34.0)	21 (45.7)	16 (59.3)	8 (34.8)
Previously treated patients, n (%)	31 (66.0)	25 (54.3)	11 (40.7)	15 (65.2)
Any interferon beta	30 (63.8)	25 (54.3)	10 (37.0)	14 (60.9)
Other MS medications	9 (19.1)	6 (13.0)	2 (7.4)	3 (13.0)

Abbreviations: *BMI* body mass index; *EDSS* Expanded Disability Status Scale; *Gd* gadolinium; *MRI* magnetic resonance imaging; *MS* multiple sclerosis; *SD* standard deviation.

### Efficacy outcomes

#### MRI outcomes

**Figure 2:**
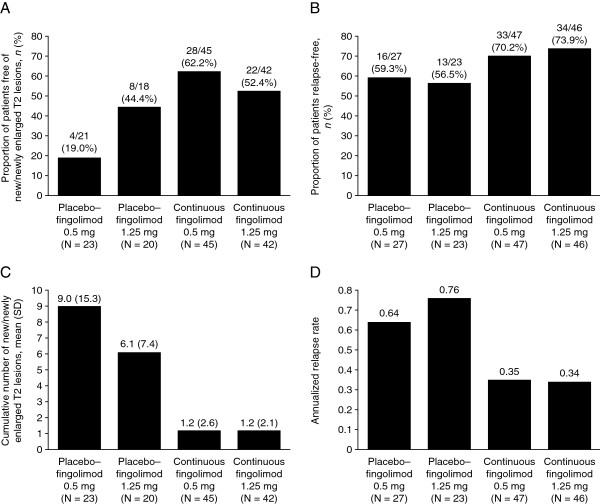
**Clinical and magnetic resonance imaging (MRI) endpoints over months 0–12.** Between-groups comparisons for **(A)** proportions of patients who were free of new/newly enlarged T2 lesions, **(B)** proportions of patients who were relapse-free (absolute number of patients free of confirmed relapses), **(C)** the cumulative number of new/newly enlarged T2 lesions and **(D)** the annualized relapse rate (calculated as total number of confirmed relapses per treatment arm divided by total number of days on the study for all patients per treatment arm, multiplied by 365.25). The MRI analysis population was used for the proportion of patients free of new/newly enlarged T2 lesions **(A)** and cumulative number of new/newly enlarged T2 lesions **(C)**. The extension full analysis set was used for the proportion of patients relapse-free **(B)** and annualized relapse rate **(D)**.

In patients switched from placebo to fingolimod therapy, inflammatory MRI activity appeared to be reduced in the extension study compared with the core phase, with apparently consistent effects across the two fingolimod doses (Table 2). The proportions of patients free of Gd-enhancing lesions at months 9 and 12 were markedly higher than those at months 3 and 6, as were the proportions of patients free of new/newly enlarged T2 lesions over months 7–12 versus months 0–6. Mean numbers of Gd-enhancing and new/newly enlarged T2 lesions were reduced in switched patients in the extension study compared with the core phase. In patients continuously treated with fingolimod, the proportions of patients free of Gd-enhancing lesions or new/newly enlarged T2 lesions remained high or increased slightly in the extension study compared with the core phase. Mean numbers of Gd-enhancing lesions decreased to zero at month 12, and the number of new/newly enlarged T2 lesions decreased over months 7–12 versus months 0–6. Over 12 months of treatment, the proportions of patients who were free of new/newly enlarged T2 lesions were higher and the mean numbers of new/newly enlarged T2 lesions were lower in the groups that received continuous fingolimod for 12 months compared with the groups that switched from placebo to fingolimod at month 6 (Figure 2, A and C).

**Table 2:** MRI and clinical endpoints in the core (months 0–6) and extension phases (months 7–12) (MRI and clinical analysis populations)

	**Continuous fingolimod groups**		**Placebo to fingolimod switch groups**	
	**Fingolimod 0.5 mg**	**Fingolimod 1.25 mg**	**Placebo-fingolimod 0.5 mg**	**Placebo-fingolimod 1.25 mg**
**MRI outcomes (MRI analysis population)**	n = 45	n = 42	n = 23	n = 20
Patients free of Gd-enhancing lesions, n/total^a^ (%)				
Both months 3 and 6	35/45 (77.8)	37/42 (88.1)	6/23 (26.1)	11/20 (55.0)
Both months 9 and 12	38/45 (84.4)	36/42 (85.7)	16/23 (69.6)	13/19 (68.4)
Number of Gd-enhancing lesions				
Month 6	0.1 (0.32)	0.2 (1.08)	1.3 (1.84)	1.2 (1.96)
Month 12	0.0 (0.15)	0.0 (0.15)	0.1 (0.31)	0.2 (0.54)
Patients free of new/newly enlarged T2 lesions, n/total^a^ (%)				
Months 0–6	30/45 (66.7)	24/42 (57.1)	5/23 (21.7)	10/19 (52.6)
Months 7–12	39/45 (86.7)	37/42 (88.1)	13/21 (61.9)	11/17 (64.7)
Number of new/newly enlarged T2 lesions				
Months 0–6	0.9 (2.1)	1.0 (2.0)	7.7 (14.5)	5.1 (6.8)
Months 7–12	0.3 (1.1)	0.2 (0.5)	0.6 (1.2)	0.5 (0.9)
**Clinical outcomes (clinical analysis population)**	n = 47	n = 46	n = 27	n = 23
Patients free of relapse, n^b^ (%)				
Months 0–6	38 (80.9)	38 (82.6)	18 (66.7)	14 (60.9)
Months 7–12	42 (89.4)	41 (89.1)	24 (88.9)	21 (91.3)
Annualized relapse rate^c^				
Months 0–6	0.47	0.39	0.97	1.22
Months 7–12	0.23	0.28	0.26	0.21
EDSS score change from core baseline				
Month 6	0.00 (0.36)	−0.21 (0.59)	0.04 (1.00)	0.26 (0.78)
Month 12	−0.02 (0.46)	−0.02 (0.83)	−0.32 (0.66)	−0.11 (0.95)

All values are means (standard deviations) unless otherwise indicated.

^a^The numerators (n) indicate the number of patients free of Gd-enhancing lesions or new/newly enlarged T2 lesions; the denominators (total) indicate the number of patients for whom MRI data were available. The percentages were calculated as n/total × 100.

^b^Absolute number of patients free of confirmed relapses.

^c^Calculated as total number of confirmed relapses per treatment arm/total number of days on the study for all patients per treatment arm × 365.25.

Abbreviations: *EDSS* Expanded Disability Status Scale; *Gd* gadolinium; *MRI* magnetic resonance imaging.

### Clinical outcomes

**Figure 3:**
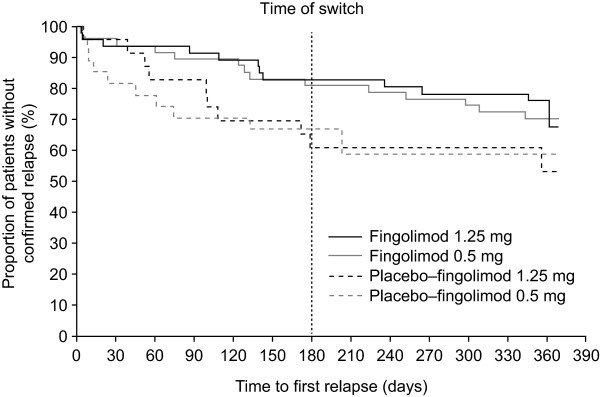
Kaplan-Meier plot of time to first confirmed relapse up to month 12 (extension full analysis set).

In individuals switched from placebo to fingolimod therapy, the proportions of patients who were free of relapse increased over months 7–12 compared with months 0–6, and ARRs over months 7–12 were markedly reduced compared with months 0–6 (Table 2). Kaplan-Meier plots demonstrated a decreased risk of relapse over months 7–12 compared with months 0–6 for those switching treatment from placebo to fingolimod (Figure 3). In patients continuously treated with fingolimod, the proportion of patients who were free of relapse remained high in the extension study compared with the core phase, while ARRs further decreased in the extension study compared with the core phase. The risk of relapse on Kaplan-Meier plots remained similar in the continuous fingolimod 1.25 mg and 0.5 mg groups over the core and extension phases (Figure 3). The proportions of patients who were free of relapse were higher, and ARRs were lower, for individuals who received continuous fingolimod therapy for 12 months compared with patients whose treatment was switched from placebo to fingolimod at month 6 (Figure 2, B and D).

### Safety and tolerability

A higher proportion of patients experienced an AE in the 1.25 mg groups relative to the 0.5 mg groups during the extension phase (months 7–12) in both continuous fingolimod and switched treatment groups (Table 3). The incidence of serious AEs was lower in the continuously treated groups than in the switched groups, and no serious AEs occurred in the continuous fingolimod 0.5 mg group during the extension phase (Table 3). The total incidence of AEs leading to study drug discontinuation was lower in the continuously treated groups than the switched groups (Table 3). No AE leading to study drug discontinuation occurred in more than one patient in each treatment group.

**Table 3:** Most frequent adverse events and serious adverse events during the extension phase (extension safety population)

**Adverse event, n (%)**	**Placebo-fingolimod 0.5 mg (n = 27)**	**Placebo-fingolimod 1.25 mg**^**a **^**(n = 23)**	**Continuous fingolimod 0.5 mg (n = 47)**	**Continuous fingolimod 1.25 mg**^**a **^**(n = 46)**
Any adverse event	22 (81.5)	23 (100)	35 (74.5)	41 (89.1)
Any serious adverse event	1 (3.7)	4 (17.4)	1 (2.1)	0 (0.0)
Any adverse event leading to discontinuation of study drug	5 (18.5)	3 (13.0)	2 (4.3)	0 (0.0)
Any infection or infestation adverse event	11 (40.7)	14 (60.9)	23 (48.9)	28 (60.9)
**Most commonly reported adverse events**^**b**^				
Nasopharyngitis	7 (25.9)	7 (30.4)	16 (34.0)	19 (41.3)
Liver function test abnormal	4 (14.8)	9 (39.1)	3 (6.4)	4 (8.7)
Leukopenia	4 (14.8)	1 (4.3)	0 (0.0)	3 (6.5)
Bradycardia	0 (0.0)	3 (13.0)	0 (0.0)	0 (0.0)
Influenza	0 (0.0)	3 (13.0)	0 (0.0)	2 (4.3)
Second-degree atrioventricular block	0 (0.0)	3 (13.0)	0 (0.0)	0 (0.0)
Headache	0 (0.0)	2 (8.7)	5 (10.6)	0 (0.0)
Lymphocyte count decreased	1 (3.7)	2 (8.7)	1 (2.1)	2 (4.3)
Lymphopenia	0 (0.0)	2 (8.7)	0 (0.0)	3 (6.5)
γ-glutamyltransferase increased	1 (3.7)	2 (8.7)	1 (2.1)	0 (0.0)
White blood cell count decreased	0 (0.0)	2 (8.7)	0 (0.0)	1 (2.2)
Eczema	0 (0.0)	1 (4.3)	1 (2.1)	4 (8.7)
Rash	2 (7.4)	1 (4.3)	1 (2.1)	0 (0.0)
Alanine aminotransferase increased	2 (7.4)	0 (0.0)	0 (0.0)	0 (0.0)
Blood triglycerides increased	2 (7.4)	0 (0.0)	0 (0.0)	0 (0.0)
Dental caries	0 (0.0)	0 (0.0)	0 (0.0)	3 (6.5)
Diarrhea	1 (3.7)	0 (0.0)	2 (4.3)	3 (6.5)
Stomatitis	1 (3.7)	1 (4.3)	0 (0.0)	3 (6.5)
Pharyngitis	0 (0.0)	1 (4.3)	1 (2.1)	3 (6.5)
Tinea pedis	1 (3.7)	1 (4.3)	3 (6.4)	0 (0.0)
Skin papilloma	0 (0.0)	0 (0.0)	3 (6.4)	0 (0.0)
**Serious adverse events**				
Bradycardia	0 (0.0)	2 (8.7)	0 (0.0)	0 (0.0)
Leukoencephalopathy^c^	0 (0.0)	1 (4.3)	0 (0.0)	0 (0.0)
Multiple sclerosis relapse	0 (0.0)	0 (0.0)	1 (2.1)	0 (0.0)
Neuromyelitis optica	1 (3.7)	0 (0.0)	0 (0.0)	0 (0.0)
Abortion induced	0 (0.0)	1 (4.3)	0 (0.0)	0 (0.0)

^a^All patients receiving fingolimod 1.25 mg/day were switched to fingolimod 0.5 mg/day after the fingolimod 1.25 mg/day dose was discontinued from all multiple sclerosis clinical studies.

^b^Adverse events reported in 5% of patients or more in any treatment group during the extension phase.

^c^This single case of leukoencephalopathy was not of the type associated with progressive multifocal leukoencephalopathy.

Infections and infestations were the most commonly reported AEs and appeared to occur in generally similar proportions of continuously treated and switched patients, but were slightly more common in the fingolimod 1.25 mg groups than the 0.5 mg groups (Table 3). No infections were reported as serious AEs (Table 3) and no infections were responsible for study drug discontinuation. Nasopharyngitis was the most commonly reported AE, occurring in 25.9–41.3% of patients. Influenza was more frequent in the placebo-fingolimod 1.25 mg switch group than in the continuous fingolimod 1.25 mg group and did not occur in the fingolimod 0.5 mg treatment groups. Herpes zoster infections occurred in 3.7% and 4.3% of switched patients (placebo-fingolimod 0.5 mg and placebo-fingolimod 1.25 mg, respectively), but were absent in both continuously treated groups.

The only recorded cardiac AEs were second-degree atrioventricular (AV) block and bradycardia, which each occurred in 3 (13%) patients in the placebo-fingolimod 1.25 mg group, and all occurred at first dose (Table 3). Of these, two of the bradycardia cases were reported as serious AEs.

Abnormal liver function tests and liver enzyme elevations occurred more frequently, and led to study drug discontinuation more frequently, in the switched patients during the extension study phase than in the continuously treated patients (Table 3). In switched patients with abnormal liver function test results, alanine aminotransferase (ALT), aspartate aminotransferase (AST) and γ-glutamyl transferase (GGT) levels were elevated from month 6.5 onwards and reached a maximum between months 9 and 12. In continuously treated patients with abnormal test results, elevations in ALT, AST and GGT were observed from 15 days after initiation of treatment in the core study: a maximum level was reached at month 3 for ALT and AST and at month 6.5 for GGT; levels of ALT, AST and GGT then remained stable up to month 12.

No deaths, malignancies or confirmed cases of macular edema were reported in patients treated continuously with fingolimod or in switched patients over months 0–12. However, a 42-year-old man in the fingolimod 0.5 mg group died approximately 1 year after discontinuing study drug (discontinuation was due to a serious MS relapse). The cause of death was diagnosed at autopsy as Epstein-Barr virus (EBV)-related B-cell lymphoma of the brain accompanying non-methotrexate-associated iatrogenic immunodeficiency-associated lymphoproliferative disorder of the lung, kidney, thyroid and jejunum (further details are provided in the Additional file 1). Although the B-cell lymphoma was diagnosed 5 months after discontinuation of study drug and after a relatively brief period of treatment with fingolimod of 9 months, a relationship between the B-cell lymphoma and the study medication cannot be excluded and was suspected by the investigator.

### Clinical courses and safety events in patients positive for anti-AQP4 antibodies

Anti-AQP4 antibody test results were obtained from one patient during the processing of a serious AE (multifocal white matter lesions in an individual in the placebo-fingolimod 1.25 mg group) and retrospectively from the medical histories of a further 67 patients. Of these 68 patients, four (5.9%) tested positive and three of these entered the extension phase. The clinical courses and safety events in these patients are described in Table 4 and Additional file 1. In two of these patients, leukoencephalopathy (multifocal white matter lesions; patient 4 in Table 4) and NMO (patient 3 in Table 4) were reported during months 7–12 as serious AEs (Table 3).

**Table 4:** Summary of clinical course and safety events in patients positive for anti-AQP4 antibodies

**Patient**	**Treatment**	**Clinical course and safety events (onset**^**a**^**)**
**Continuous fingolimod**		
1	Fingolimod 0.5 mg	Bradycardia (day 2); chest discomfort (day 2); enlargement of a pre-existing spinal cord lesion, LESCL, pons and midbrain lesions (day 9); myelitis and LESCL (day 145); diplopia, right upper limb paresis, multiple lesions in the cervical and thoracic cord, brainstem, cerebellum and cerebrum (day 235)
2^b^	Fingolimod 1.25 mg	Decreased heart rate (day 1); myelitis relapse (day 5); decreased heart rate (day 5; day 1 after drug interruption); abnormal liver function test result (day 85); asymptomatic multiple enhancing lesions in the bilateral cerebral white matter and an asymptomatic enhancing spinal cord lesion at C6 (6 days after fingolimod discontinuation); conduction and amnestic aphasia (3 weeks after fingolimod discontinuation)
**Switched from placebo to fingolimod**		
3	Placebo/fingolimod 0.5 mg	Recurrence of right optic neuritis and myelitis (day 29 of extension phase); dysarthria, right ataxia and weakness, right frontal lobe lesion (3 months); right upper limb weakness (4 months); left parietal lobe lesion, left paresthesia, dizziness and eye movement disturbance (1 month after fingolimod discontinuation)
4	Placebo/fingolimod 1.25 mg	Relapse showing left hemispheric symptoms and exacerbation of cerebral and cerebellar white matter lesions (day 9 in the extension phase)^c^

^a^Days after initiation of fingolimod in core study or extension phase.

^b^This patient discontinued from the study on day 78 during the core phase.

^c^This case was not diagnosed as progressive multifocal leukoencephalopathy.

Abbreviations: *AQP4* aquaporin 4; *LESCL* longitudinally extensive spinal cord lesion.

## Discussion

This extension study provides additional data with which to establish the efficacy and safety profile of fingolimod in Japanese patients with relapsing MS. Overall, the efficacy and safety findings were consistent with those in the core study [10]. Patients who received continuous fingolimod treatment for 12 months experienced sustained or improved efficacy outcomes during the extension phase and had reduced relapse rates and MRI lesion activity compared with those in the core study. Furthermore, efficacy endpoints were consistently superior at month 12 in the continuous fingolimod groups compared with the placebo-fingolimod groups.

The results of this trial are consistent with those of previously reported clinical studies of fingolimod in predominantly Caucasian populations [6-8,14]. The results are clinically important because there are genetic, metabolic and lifestyle differences between Caucasian and Japanese populations [15,16], as well as differences in the presentation of MS that may affect response to therapy. It has been postulated that the higher prevalence of optic-spinal MS (OSMS) in Japan versus Western countries is due to a higher prevalence of NMO, which is also a demyelinating disease with some overlapping symptoms to MS and is characterized by LESCLs [10]. Historically, no distinction was made between MS and NMO. However, debate continues about whether OSMS is actually a distinct disease from both MS and NMO because many Japanese patients who present with OSMS are seronegative for anti-AQP4 (a marker for NMO) and remain free from LESCLs [17]. This study was designed to exclude NMO patients by using the presence of LESCLs on spinal MRI as exclusion criteria at screening. Patients were eligible for the study if the diagnosis of MS was confirmed by the study investigators and if they fulfilled MRI criteria. There was no exclusion criterion related to the presence of anti-AQP4 antibodies. The anti-AQP4 antibody test results were collected from medical records retrospectively for patients who consented to provide the data upon occurrence of a severe adverse event.

In both the continuous fingolimod and placebo-fingolimod groups, the safety profile was consistent with that seen in previous clinical studies [6,8,14,18]. No new AEs, deaths, or fatal or serious infections were reported during the extension phase. The cardiac AEs reported in the placebo-fingolimod groups (transient asymptomatic bradycardia and second-degree AV block) were consistent with those observed in the core study and in global clinical studies [6-8,14], and have been shown to be an expected consequence of the interaction of fingolimod with S1PRs on atrial myocytes [5,19]. Liver enzyme elevations were more common in the switch groups than in the continuous fingolimod groups, consistent with the early onset and transient nature of transaminase elevations with fingolimod [6,8,14]. Global fingolimod studies have identified macular edema [6-8] as an AE of low frequency but of particular interest. In this relatively small study, specific safety monitoring did not identify any cases of macular edema up to month 12. Three cases of lymphoma, including the case of EBV-positive B-cell lymphoma described above, have been reported in the overall fingolimod clinical development program [20]. This is in line with epidemiological data reporting the background incidence of lymphoma (19.1 cases per 100,000 person-years [21]). However, because of the low incidence of malignancies and the limited duration of exposure, firm conclusions on an increased risk of malignancies with fingolimod treatment cannot be drawn yet.

Although it was reported that positivity for antibodies to AQP4 and evidence of LESCLs distinguish NMO from MS with 76% and 98% sensitivity, respectively [22], 4/68 (5.9%) patients in this extension study, which excluded individuals with LESCLs, were anti-AQP4 antibody-positive. It is therefore possible that not all cases of NMO were excluded. Exacerbation of NMO has been reported in Japanese patients treated with interferon beta for MS who were subsequently found to be positive for antibodies to AQP4 [23]. In addition, a case study of a patient who developed extensive brain lesions 14 days after initiation of fingolimod 0.5 mg in the phase 3 TRANSFORMS (Trial Assessing Injectable Interferon versus FTY720 Oral in Relapsing-Remitting Multiple Sclerosis) has been published [24]. After this patient tested positive for anti-AQP4 antibodies, the initial diagnosis of MS was changed to NMO.

In the present study, three individuals positive for antibodies to AQP4 (patients 1, 2 and 4 from Table 4) developed relapses very rapidly after introduction of either dose of fingolimod (2, 5 and 9 days after, respectively). This is in accordance with the time-course of exacerbation in the previous case report of NMO during fingolimod therapy [24]. In the core study, the proportion of relapses that occurred in the 2 weeks after study entry out of the total number of relapses was relatively low (30%, 17% and 27% for the fingolimod 1.25 mg, 0.5 mg and placebo groups, respectively). Thus, although causality cannot be established from this retrospective analysis, it may be speculated that the relapses within the first 2 weeks following fingolimod initiation in patients positive for antibodies to AQP4 represented exacerbation of disease due to the temporal sequence of events here and in the previous case report of NMO [24]. A fourth anti-AQP4-positive patient (patient 3 from Table 4) presented with three relapses during 129 days of fingolimod therapy; however, this individual was considered to have high disease activity as indicated by additional relapses before and after fingolimod therapy. The frequent relapses during fingolimod therapy in patient 3 may also be partly attributable to exacerbation of relapse. In all anti-AQP4-positive patients, the nature of relapses developed after commencing fingolimod appeared to be similar to previous relapses (myelitis in patients 1, 2 and 3, and multifocal cerebral and cerebellar white matter lesions in patient 4). Accordingly, it is conceivable that fingolimod did not alter the nature of the disease; rather, it might have enhanced the original disease activity through an unknown mechanism. In the present study, corticosteroid pulse therapy appeared to alleviate the exacerbations that emerged after fingolimod initiation, leaving little or no residual effects (except in patient 4, who had moderate residual disability despite intravenous immunoglobulin administration in addition to steroid pulse therapy), although these observations on corticosteroid effects did not have a control for comparison. Together, the present findings suggest that caution is needed if introducing fingolimod in patients with idiopathic central nervous system demyelinating disease with atypical MS features without testing for anti-AQP4 antibodies first.

The limitations of this observational extension study include the need to switch treatment for all patients receiving fingolimod 1.25 mg to 0.5 mg and the small sample size, which prevented meaningful statistical interpretation – while larger cohorts would be desirable, the low incidence of MS in Japan presents a significant recruitment challenge.

## Conclusion

Continuous fingolimod treatment for MS over 12 months is associated with consistent efficacy outcomes with no deterioration in the predictable and manageable safety profile.

## Abbreviations

AE: Adverse event; ALT: Alanine aminotransferase; AQP4: Aquaporin-4; ARR: Annualized relapse rate; AST: Aspartate aminotransferase; AV: Atrioventricular; EBV: Epstein-Barr virus; EDSS: Expanded Disability Status Scale; Gd: Gadolinium; GGT: γ-glutamyl transferase; LESCL: Longitudinally extensive spinal cord lesion; MRI: Magnetic resonance imaging; MS: Multiple sclerosis; NMO: Neuromyelitis optica; OSMS: Optic-spinal multiple sclerosis; RRMS: Relapsing-remitting multiple sclerosis; S1PR: Sphingosine 1-phosphate receptor; TRANSFORMS: Trial Assessing Injectable Interferon versus FTY720 Oral in Relapsing-Remitting Multiple Sclerosis.

## Competing interests

Fingolimod is under clinical development by the study sponsor, Novartis Pharma KK and Mitsubishi Tanabe Pharma Corporation, Tokyo, Japan. TK, IT, PvR and LZ-A are employees of Novartis and KN is an employee of Mitsubishi Tanabe Pharma Corporation.

## Authors’ contributions

All authors participated in the design of the study, and read and approved the final manuscript. IT led the statistical analyses. QH conducted the magnetic resonance imaging.

## Pre-publication history

The pre-publication history for this paper can be accessed here:

http://www.biomedcentral.com/1471-2377/14/21/prepub

## Supplementary Material

Additional file 1 Additional results, including the clinical course and safety events in patients positive for AQP4 antibodies. Click here for file
