# Supplementary material for: Fingolimod (FTY720) therapy in Japanese patients with relapsing multiple sclerosis over 12 months: results of a phase 2 observational extension
Source: BMC Neurol. 2014 Jan 29;14:21. doi: 10.1186/1471-2377-14-21 (PMC3911956; doi:10.1186/1471-2377-14-21)
Supplement: Additional file 1 — Additional results, including the clinical course and safety events in patients positive for AQP4 antibodies. [file 1471-2377-14-21-S1.pdf]

Additional file 1

Methods (not reported in main manuscript)

Eligibility criteria

Eligible patients had a relapsing-remitting (one or more confirmed relapses in the previous year or two or more in the previous 2 years) or secondary progressive course, or at least one gadolinium (Gd)-enhancing T1-weighted brain lesion within the 30 days prior to study commencement. Participants also had to have at least one T2-weighted brain lesion and a score of 0-6.0 on the Expanded Disability Status Scale (EDSS) [1].

Study design

All patients who had yet to enter the extension study and who had received fingolimod 1.25 mg or placebo in the core study, and patients who were continuing to receive fingolimod 1.25 mg in the extension study, had their treatment switched to fingolimod 0.5 mg. From the date of the implementation of this protocol amendment, the study continued with an open-label design. The protocol amendment resulted in 19 of 46 patients in the fingolimod 1.25 mg continuous group and 5 of 23 patients in the placebo-fingolimod 1.25 mg group having their therapy switched to fingolimod 0.5 mg before month 12.

Results (not reported in main manuscript)

Laboratory evaluations

As observed in the core study, mean absolute lymphocyte counts were reduced at day 15 of the extension in patients whose treatment was switched from placebo to fingolimod

(0.48 × 10^9/L [28.4% of the core study baseline]). The reduced lymphocyte counts remained stable, with a mean absolute lymphocyte count of 0.50 × 10^9/L (30.6% of the core study baseline) at month 12 and 82% of patients maintained a lymphocyte count > 0.2 × 10^9/L throughout months 7-12. In continuously treated patients, counts remained stable throughout the extension phase, with corresponding mean absolute lymphocyte counts of 0.44 × 10^9/L (25.3% of baseline) and 0.43 × 10^9/L (24.4% of baseline) at month 12: the percentage of patients with a lymphocyte count < 0.2 × 10^9/L was slightly lower in months 7-12 compared with months 0-6 (17.4% versus 20.4% for 1.25 mg; 8.5% versus 8.9% for 0.5 mg, respectively). A lymphocyte count below 0.1 × 10^9/L was seen in one patient (2.2%) continuously treated with fingolimod 1.25 mg during months 7-12.

Death

A 42-year-old man in the fingolimod 0.5 mg group died approximately 1 year after discontinuing study drug (discontinuation was due to a serious multiple sclerosis [MS] relapse). A chest X-ray taken 5.5 months after the last dose of study medication revealed a suspected malignancy. Clinical diagnosis prior to autopsy was MS and lymphoma, as a cutaneous T-cell lymphoma had been diagnosed and a biopsy had identified a suspected Epstein-Barr virus (EBV)-related lymphoproliferative disorder of the kidney. At autopsy, diffuse B-cell lymphoma of the brain was confirmed, although no clear MS lesions were present. The pathologist diagnosed this case as an EBV-related diffuse B-cell lymphoma of the brain accompanying non-methotrexate-associated iatrogenic immunodeficiency-associated lymphoproliferative disorder of the lung, kidney, thyroid and jejunum.

Clinical course and safety events in patients positive for AQP4 antibodies

Four cases of aquaporin 4 (AQP4) antibody-positive patients were identified during the study either via their medical history collected retrospectively or during the processing of a serious adverse event (AE) (see Table 5, main manuscript).

Patient 1 (Table 4, main manuscript, Supplementary Figure 1A), a 50-year-old woman, had a history of six relapses and interferon β-1b treatment. Four months before entering the trial, a pre-existing spinal cord lesion was found in thoracic segments 4-6 with symptomatic low back pain. She experienced bradycardia and chest discomfort on day 2 after initiation of fingolimod 0.5 mg in the core study, for which she was hospitalized for on day 4 (heart rate: 44 beats per minute [bpm]); these events resolved after a period of rest. Over the next 5 days, she experienced several episodes of anterior chest pain. Spinal cord magnetic resonance imaging (MRI) on day 9 after fingolimod initiation revealed enlargement of the pre-existing spinal cord lesions in thoracic segments 6-8. Brain MRI showed a new T2 lesion at the bilateral basilar part of the pons and a nodular-shaped high T2 lesion in the right cerebral peduncle of the midbrain reaching the tegmentum and thalamus. Corticosteroid pulse therapy resolved the patient's symptoms. She temporarily discontinued fingolimod for 1 month and then resumed fingolimod treatment. On day 146 of the core study, the patient experienced a second relapse with cervical pain and hypoesthesia of right upper and lower extremities; at this time, spinal cord MRI showed LESCLs in cervical segments 2-6 and the patient had an EDSS score of 5.0. Fingolimod treatment was subsequently discontinued owing to relapse on day 72 of the extension phase, with the patient experiencing unpleasant pharyngeal sensation, diplopia and right paralysis of the upper extremities including the face. Brain MRI revealed multiple cerebral and cerebellar lesions, extending to the brainstem, and spinal cord MRI showed lesions in cervical segments 5-6 and thoracic segments 3-4. EDSS scores were 2.5 at baseline, 1.5 at month 3 and 2.0 at month 6. The numbers of Gd-

enhancing and T2 lesions at baseline were 0 and 4, respectively; during the trial, numbers of Gd-enhancing and new T2 lesions were 1 and 10, respectively, at 1 month, and 0 and 1, respectively, at 6 months, indicating that asymptomatic brain lesions appeared during the trial.

Patient 2 (Table 4, main manuscript, Supplementary Figure 1B), a 54-year-old woman, had a history of recurrent spinal cord involvement and a Gd-enhancing lesion of 1.5-vertebral segments in length at the thoracic 2-3 segment level at the second relapse. Five days after commencing fingolimod 1.25 mg, she developed left leg weakness. Neurological examination revealed left side-dominant paraparesis, hypesthesia below the bilateral lumbar 1 level and hypalgesia/thermo hypesthesia below the right lumbar 1 level; the patient had an EDSS score of 7.0. MRI showed a Gd-enhancing lesion of 1.5 vertebral segments in length at the thoracic 4-5 level. Subsequent MRI at 21 days after initiation of fingolimod revealed longitudinally extensive spinal cord lesions (LESCLs) at the thoracic 2-7 level. The patient’s symptoms disappeared after corticosteroid pulse therapy. Fingolimod was permanently discontinued after approximately 3 months owing to elevated liver transaminases. Four days later, the patient developed multiple, asymptomatic, cloud-like, Gd-enhancing lesions in the bilateral cerebral white matter following fever (38°C), and 6 days after fingolimod discontinuation she developed an asymptomatic Gd-enhancing spinal cord lesion at cervical level 6. Twenty-seven days after fingolimod discontinuation, conduction and amnestic aphasia occurred with the appearance of Gd-enhancing lesions in the left temporal lobe. These lesions and the aphasia disappeared following corticosteroid pulse therapy. EDSS scores were 1.5 at core study baseline, 5.5 at the time of fingolimod discontinuation (day 85) and 4.0 at the final follow-up examination (day 155). The numbers of Gd-enhancing and T2 lesions were 0 and 2, respectively, at baseline, 16 and 34, respectively, at the time of fingolimod

discontinuation, and 2 and 5, respectively, at the final follow-up examination (day 155).

Patient 3 (Table 4, main manuscript, Supplementary Figure 1C), a 38-year-old woman, was diagnosed with MS 4 years before enrollment in the trial – she had had six relapses, including one in the year before study entry. The patient was enrolled in the placebo arm in the core study and switched to fingolimod 0.5 mg in the extension phase. Numbers of Gd-enhancing and T2 lesions at baseline were 0 and 4, respectively, and 0 and 0, respectively at both 3 and 6 months of the core study. Baseline EDSS score was 1.5, which increased to 5.0 at months 3 and 6 owing to severe right optic neuritis at month 2 of the core study. On day 29 of fingolimod 0.5 mg treatment, the patient developed enlargement of a right visual field defect and sensory deficit in the left upper limb and trunk; an MRI examination revealed LESCLs at cervical segments 2-7 and a resulting test for AQP4 antibodies was positive. Corticosteroid pulse therapy relieved the patient's symptoms. However, 3 months after entry into the extension phase, the patient experienced a relapse, which presented as left upper limb weakness and ataxia, and dysarthria. At this time, new lesions were evident in the right frontal lobe, and bilateral corpus callosum and cingulate gyri. Corticosteroid pulse therapy again ameliorated her symptoms. One month later (4 months after the entry into the extension phase), the patient developed right upper limb weakness and new lesions in the right medulla, right temporal lobe and left parietal lobe. The patient was successfully treated with immunoadsorption. Fingolimod was discontinued after approximately 4 months (day 129) of treatment. About 1 month after fingolimod discontinuation, the patient had a further relapse with left side paresthesia, dizziness and right eye movement disturbance. These symptoms were alleviated by steroid pulse therapy and azathioprine, and no residual effects were recorded, except for persistent right optic nerve damage.

Patient 4 (Table 4, main manuscript, Supplementary Figure 1D), a 48-year-old woman, was diagnosed with MS approximately 3 years before entering the trial. The diagnosis was based on evidence of multiple cerebral white matter lesions; however, an AQP4 antibody test performed at that time was positive. Before entering the study, the patient experienced a relapse with a mild disturbance of consciousness and left facial and upper limb weakness. MRI showed multiple high signal areas on T2-weighted images across a wide expanse in the cerebral white matter. Fluid attenuated inversion recovery (FLAIR) images revealed a large lesion of 3 cm in size in the right posterior temporal lobe and one irregularly shaped Gd-enhancing area 2 cm in diameter in the right frontal lobe white matter. The patient was treated with two courses of corticosteroid pulse therapy. The patient was enrolled to the placebo arm in the core study and switched to fingolimod 1.25 mg in the extension phase. During the study screening period, an independent review of baseline MRIs found multiple areas of nodular-shaped, high signal T2 lesions in the midbrain, cerebellar peduncles and bilateral subcortical white matter, and around the lateral ventricles, including one large lesion with a necrotic tendency in the right posterior temporal lobe (Supplementary Figure 2A). In the core study, EDSS scores were 2.5 at baseline, 2.5 at month 3 and 2.0 at month 6. Numbers of Gd-enhancing and T2 lesions were 0 and 71, respectively, at baseline, and 0 and 0, respectively, at the 3, 4, 5 and 6 month time points of the core study. Nine days after initiation of fingolimod 1.25 mg in the extension phase, the patient experienced an intermittent headache. Two days later, she developed a disturbance of consciousness, aphasia, apraxia, right hemi-spatial neglect and right upper limb weakness. MRI revealed high signal T2 lesions bilaterally in the cerebellum and around the cerebral ventricles, and a large subcortical lesion was detected in the left temporal lobe (Supplementary Figure 2B). Three months after fingolimod initiation, the patient was improving clinically (apart from apraxia,

acalculia, disorientation, recent memory disturbance and motor perseveration), and there was partial resolution of MRI lesions.

References

1. Kurtzke JF: **Rating neurologic impairment in multiple sclerosis: an expanded disability status scale (EDSS).** *Neurology* 1983; **33**:1444–1452.

Supplementary figures

Supplementary Figure 1. Clinical courses of four AQP4 antibody-positive patients, patients 1 (A), 2 (B), 3 (C), and 4 (D), before study entry and during the core study and extension phase.

A

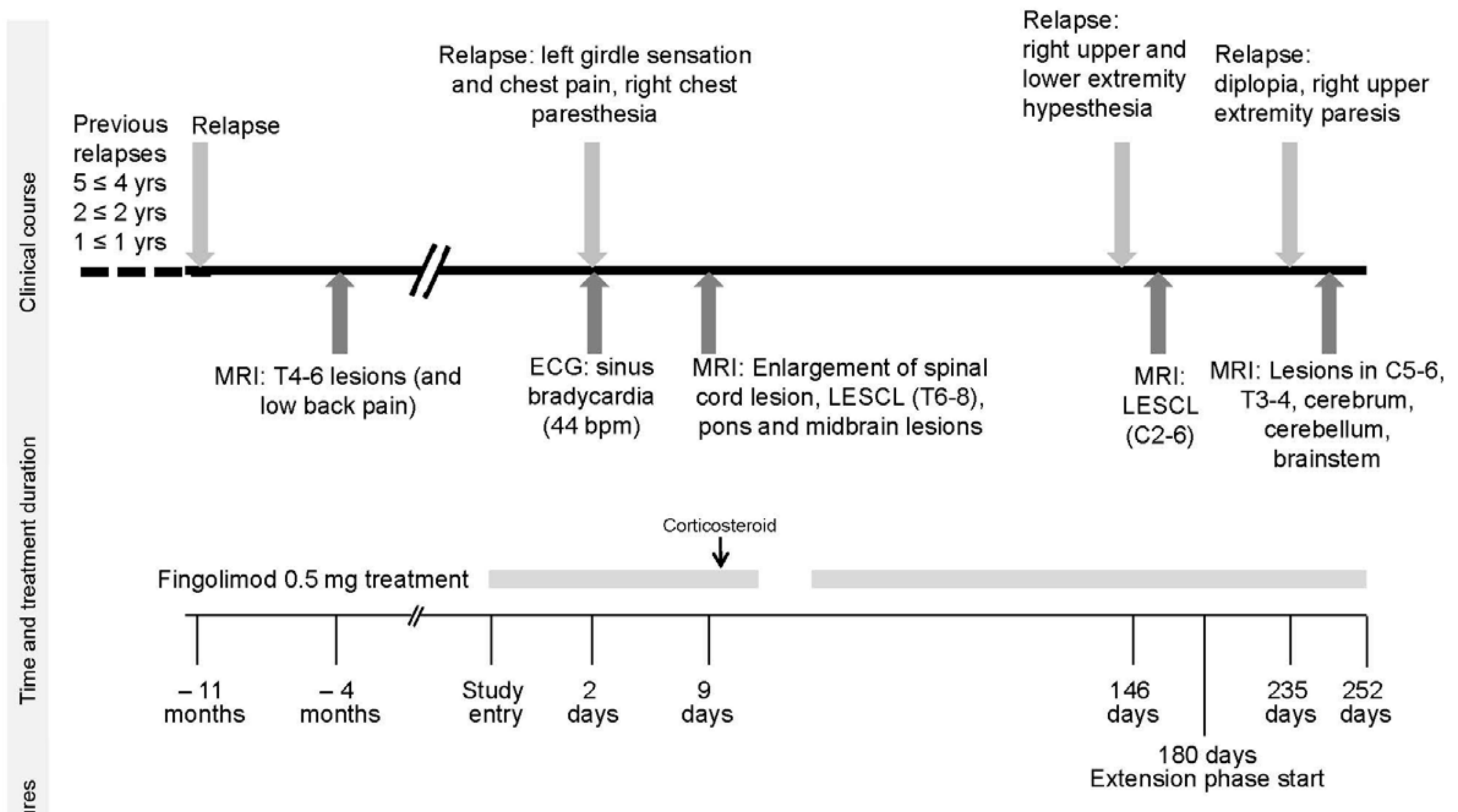

Clinical and MRI measures

	**Study baseline**	**9 days**	**3 months**	**146 days**	**6 months**	**235 days**
EDSS	2.5	3.0	1.5	5.0	2.0	5.0
Gd-enhancing	0		1		0	
T2	4					
New T2			10		1	

B

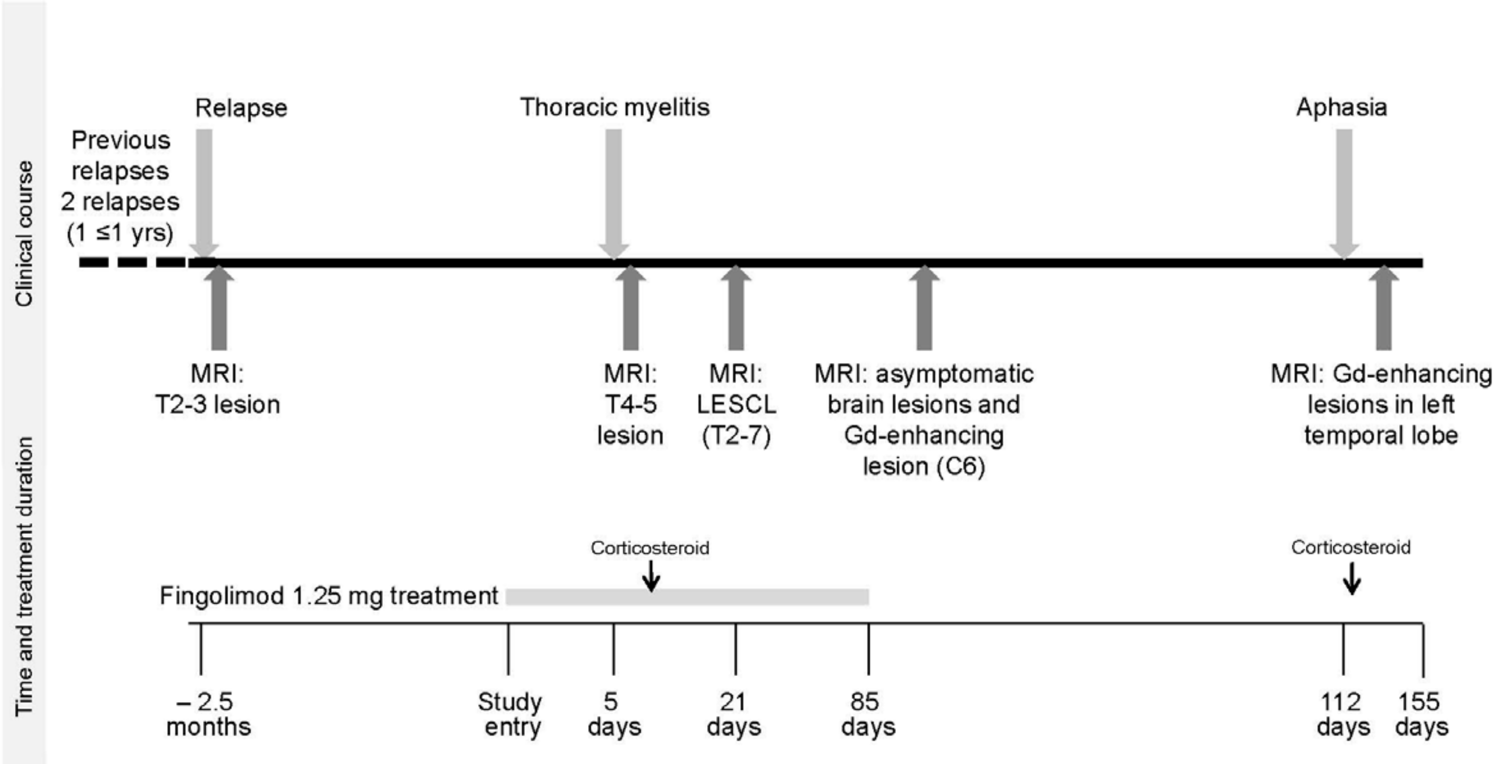

	**Study baseline**	**6 days**	**85 days**	**155 days**
EDSS	1.5	7.0	5.5	4.0
Gd-enhancing	0		16	2
T2	2			
New T2			34	5

C

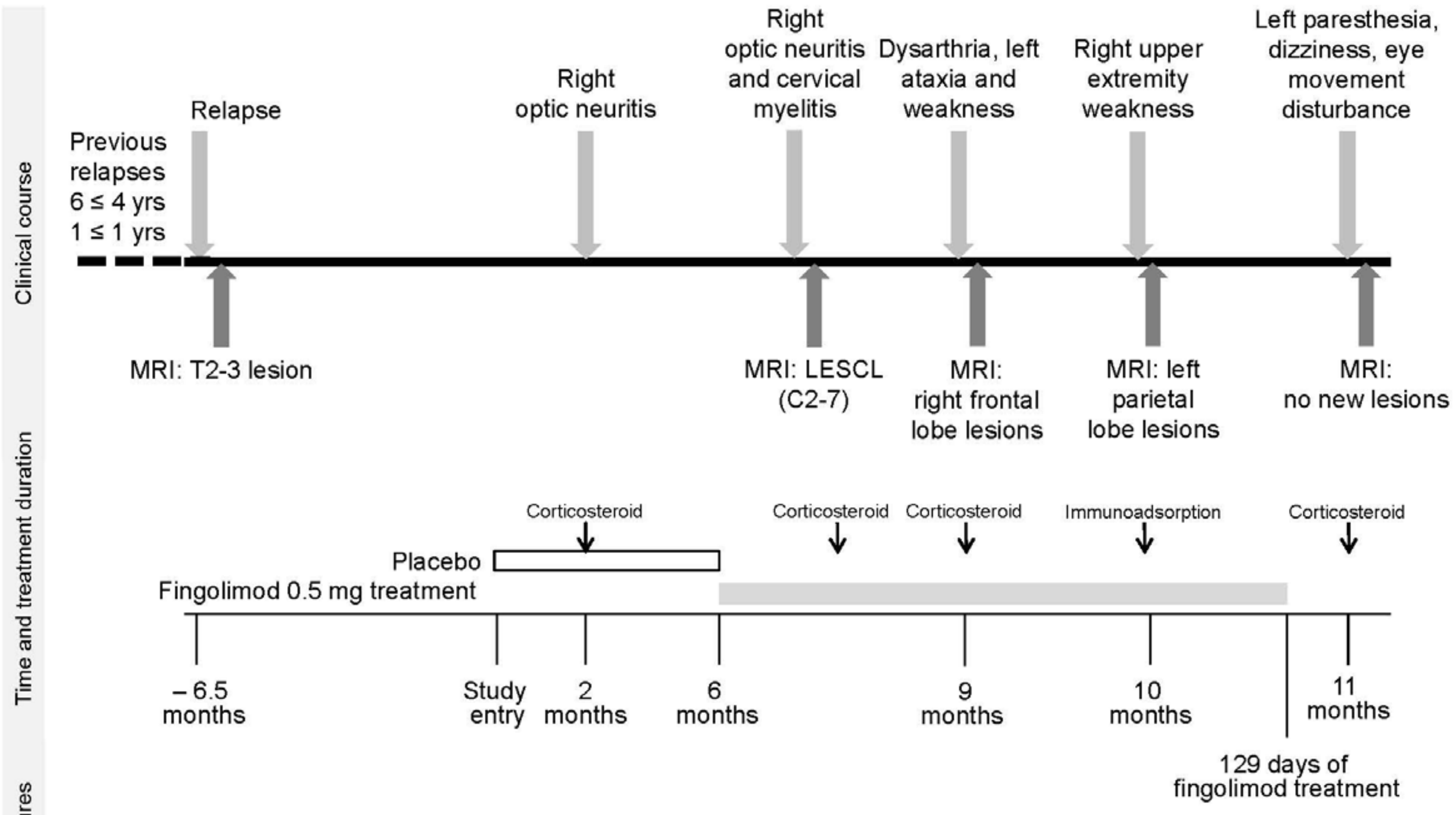

	Study baseline	3 months	6 months
EDSS	1.5	5.0	5.0
Gd-enhancing	0	0	0
T2	4		
New T2		0	0

D

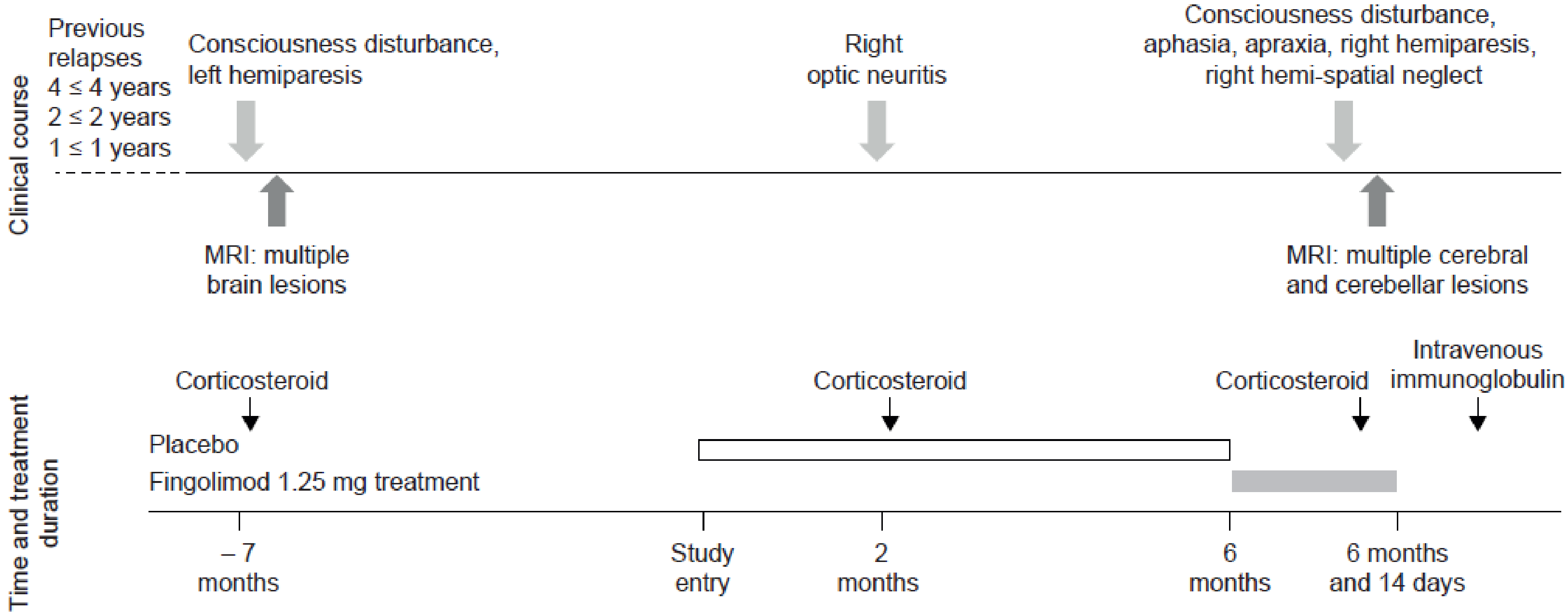

Clinical and MRI measures

	**Study baseline**	**3 months**	**6 months**
EDSS	2.5	2.5	2.0
Gd-enhancing	0	0	0
T2	71		
New T2		0	0

Patient 1 discontinued fingolimod on day 252 (extension day 72) owing to relapse. Patient 2 discontinued on day 85 owing to liver dysfunction. Patient 3 discontinued after 129 days of fingolimod treatment owing to unsatisfactory therapeutic effect. Patient 4 discontinued on day 14 in extension phase owing to leukoencephalopathy.

Abbreviations: C = cervical spinal cord; bpm, beats per minute; ECG = electrocardiogram; EDSS = Enhanced Disability Status Scale; Gd = gadolinium; LESCL,= longitudinally extensive spinal cord lesions; MRI = magnetic resonance imaging; T = thoracic spinal cord.

Supplementary Figure 2. Magnetic resonance imaging (MRI) findings from an aquaporin-4 antibody-positive patient (placebo-fingolimod 1.25 mg group, patient 4 in Table 5) with multifocal white matter lesions. (A) Baseline MRI with punctiform and fused subcortical lesions, one of which formed a large cavity-like lesion in the right posterior temporal and parietal lobes. **(B)** MRI at 19 days after initiation of fingolimod showing large new lesions in the left frontal lobe and bilateral cerebellar hemispheres.

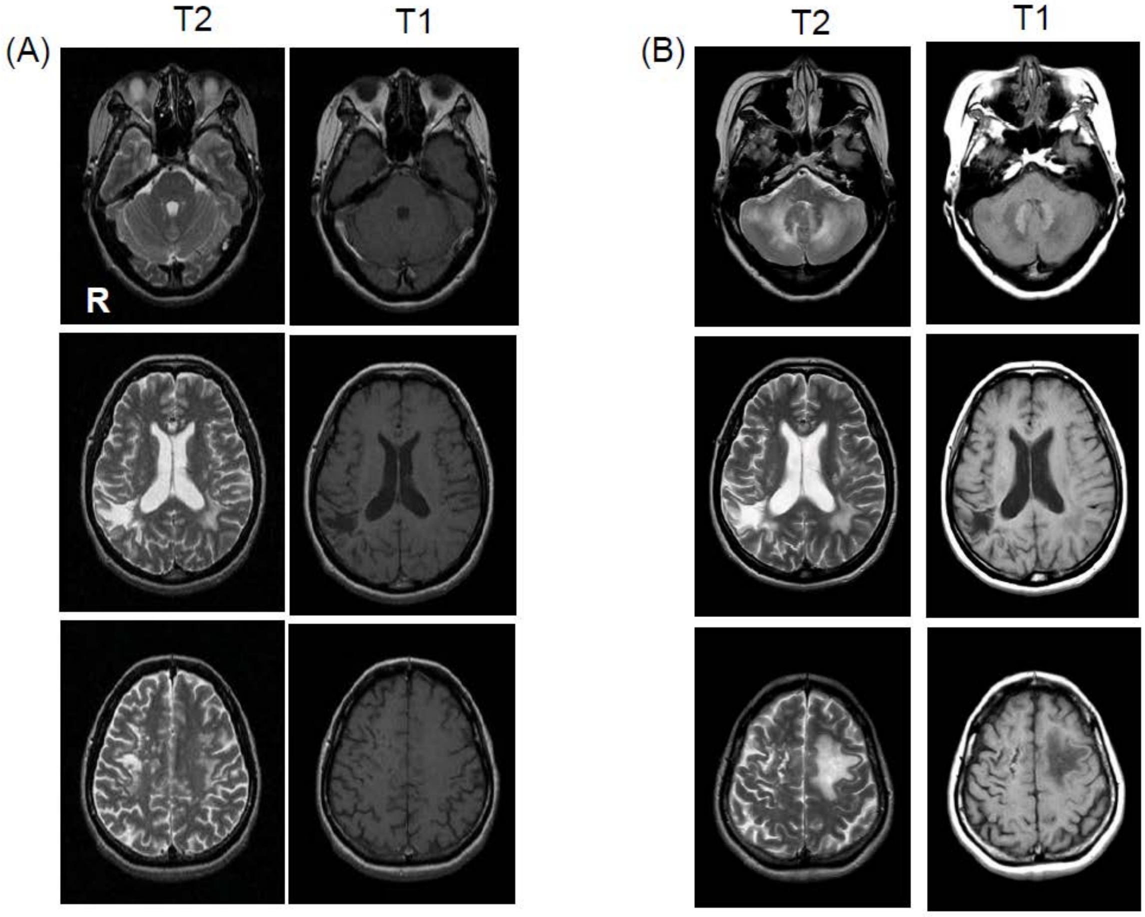

Abbreviations: R = right side; T1 = T1-weighted MRI image; T2 = T2-weighted MRI image.
